# Diseases of the Gums

**Published:** 1842-03

**Authors:** 


*Diseases of the Gums.*—In the present number of the Journal, will be found an article on "conjoined suppuration of the gums," by Dr. H. H. Hayden, whose request with regard to the publication of a paper on the same disease, but denominated the "devastating process," by Dr. Koecker, we shall with pleasure comply with. Dr. Koecker's views upon the subject, as contained in the paper here alluded to, will appear in the regular course of the publication of his "Principles of Dental Surgery," in the library part of our next number. From the extensive experience and well known professional abilities of both these gentlemen, we doubt not that what they have said upon this highly important and interesting subject will be read with much interest. Their opinions are certainly entitled to high consideration, and although they do not altogether agree concerning the curability of the disease, they are both evidently well acquainted with its pathology.

